# Modelling the dynamic interaction of systemic inflammation and the hypothalamic–pituitary–adrenal (HPA) axis during and after cardiac surgery

**DOI:** 10.1098/rsif.2021.0925

**Published:** 2022-04-27

**Authors:** Daniel Galvis, Eder Zavala, Jamie J. Walker, Thomas Upton, Stafford L. Lightman, Gianni D. Angelini, Jon Evans, Chris A. Rogers, Kirsty Phillips, Ben Gibbison

**Affiliations:** ^1^ Centre for Systems Modelling and Quantitative Biomedicine (SMQB), University of Birmingham, Edgbaston B15 2TT, UK; ^2^ Henry Wellcome Laboratories for Integrative Neuroscience and Endocrinology, University of Bristol, Bristol BS1 3NY, UK; ^3^ Bristol Heart Institute, Bristol Medical School, University of Bristol, Bristol BS1 3NY, UK; ^4^ College of Engineering, Mathematics and Physical Sciences, University of Exeter, Exeter EX4 4QD, UK; ^5^ Bristol Trials Centre, Bristol Medical School, University of Bristol, Bristol BS2 8HW, UK; ^6^ Department of Anaesthesia, Bristol Medical School, University of Bristol, Bristol BS2 8HW, UK; ^6^ Department of Pathology, University Hospitals Bristol NHS Foundation Trust, Bristol BS2 8HW, UK

**Keywords:** cardiac surgery, cortisol, HPA axis, acute inflammation, mathematical modelling

## Abstract

Major surgery and critical illness produce a potentially life-threatening systemic inflammatory response. The hypothalamic–pituitary–adrenal (HPA) axis is one of the key physiological systems that counterbalances this systemic inflammation through changes in adrenocorticotrophic hormone (ACTH) and cortisol. These hormones normally exhibit highly correlated ultradian pulsatility with an amplitude modulated by circadian processes. However, these dynamics are disrupted by major surgery and critical illness. In this work, we characterize the inflammatory, ACTH and cortisol responses of patients undergoing cardiac surgery and show that the HPA axis response can be classified into one of three phenotypes: single-pulse, two-pulse and multiple-pulse dynamics. We develop a mathematical model of cortisol secretion and metabolism that predicts the physiological mechanisms responsible for these different phenotypes. We show that the effects of inflammatory mediators are important only in the single-pulse pattern in which normal pulsatility is lost—suggesting that this phenotype could be indicative of the greatest inflammatory response. Investigating whether and how these phenotypes are correlated with clinical outcomes will be critical to patient prognosis and designing interventions to improve recovery.

## 1. Introduction

Major surgery and critical illness elicit a systemic inflammatory response [1], which when uncontrolled leads to major morbidity and/or death [2,3]. One of the key physiological systems that regulate the inflammatory response in humans is the hypothalamic–pituitary–adrenal (HPA) axis, which controls the pulsatile secretion of adrenocorticotrophic hormone (ACTH) and cortisol. The dynamics of these hormones are changed following major surgery [4] and critical illness [5]. Many of the models for the HPA axis and inflammation used in clinical and scientific practice are based on linear, unidirectional relationships [6,7], yet both inflammation and the HPA axis are cascades, with individual components having multiple sites of action in other systems and feedback on their own [8]. The secretion and effects of these hormones are also nonlinear, making the overall effect at the level of an organism difficult to elucidate.

**Figure 1.**
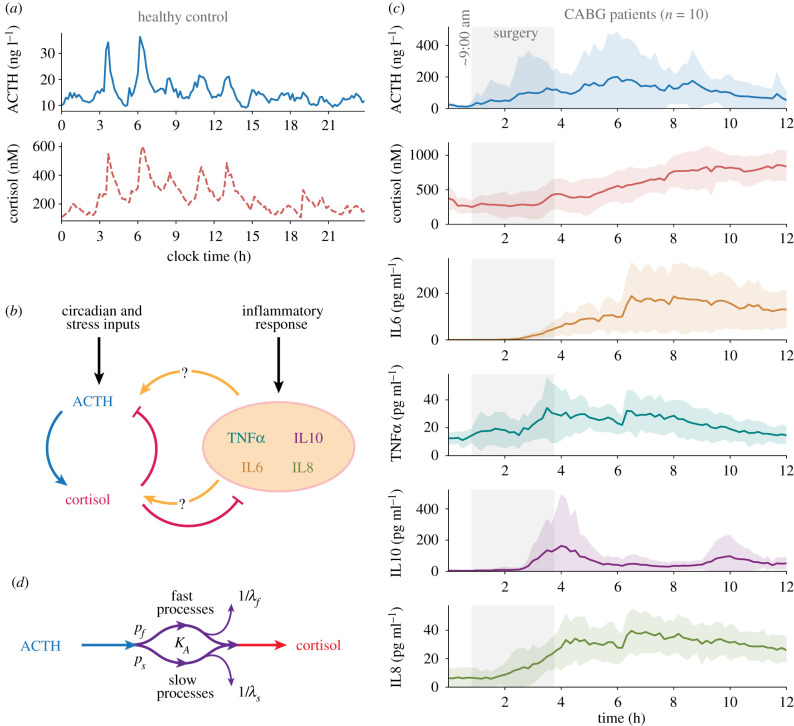
Dynamic stress and inflammatory responses following CABG. (*a*) Twenty-four hour ACTH and cortisol dynamic profile in a healthy control [4]. (*b*) Systems-level interactions between the HPA axis and inflammatory mediators. (*c*) Twelve hour dynamic profile (*μ* ± *σ*, *n* = 10) of ACTH, cortisol and inflammatory mediators IL6, TNF*α*, IL10 and IL8 during and after CABG (IL2, IL4 and IL1*α* were assayed but inconsistently detected across patients). Grey-shaded areas indicate the mean time span of CABG surgery. Detailed individual profiles are shown in electronic supplementary material, figure S2. (*d*) Schematic illustrating the mathematical model. Eleven hours of ACTH data were used as input into fast and slow cortisol activity compartments. The model was calibrated to healthy controls, and its predictions compared against patient data.

Under normal physiological conditions, ACTH and cortisol are in a state of continuous dynamic equilibration [9] and exhibit highly correlated ultradian pulsatility with an amplitude modulated by circadian processes (figure 1*a*). We have previously shown that these correlated patterns are disrupted by both surgery [4] and critical illness [10]. The most likely mediators of the interaction between inflammation and the HPA axis are cytokines such as IL1*α*, IL6 and TNF*α* and cortisol. To address the question of how the inflammatory and HPA axis responses interact at a systems level (figure 1*b*), we performed high-frequency serial blood sampling during and after coronary artery bypass grafting (CABG) surgery to generate profiles of ACTH, cortisol and inflammatory mediators (figure 1*c*). These profiles were statistically classified according to dynamic phenotype and used to calibrate a mathematical model to characterize the underlying changes in HPA physiology (figure 1*d*).

## 2. Material and methods

The study was reviewed and approved by the UK National Research Ethics Service (NRES, ref.: 11/H0107/9) and Health Research Authority (HRA). Female sex hormone cycles are known to affect the HPA axis [11,12] and for this reason, female participants were excluded.

### 2.1. Subjects: patients

Ten patients were recruited prior to surgery with informed consent from a single cardiac surgical centre and met the following inclusion criteria: male, aged 18–80 years undergoing first time, elective CABG carried out via median sternotomy. Patients were excluded if they met any of the following criteria: emergency operation, previous sternotomy, myocardial infarction within the last month, concomitant procedure with CABG, left ventricular ejection fraction less than 30%, operation to be carried out by other incision than median sternotomy (e.g. left thoracotomy), use of exogenous corticosteroids (including inhalers), history of adrenal or pituitary disease.

### 2.2. Subjects: healthy controls

Healthy controls were taken from previously published studies on healthy subjects [10,13]. In brief, healthy males were recruited with informed consent. Participants were excluded if they had recent (less than 10 days) trans-meridian travel and if they were exposed to exogenous glucocorticoids in the previous three months. Participants maintained conventional work and sleeping patterns and were sampled from peripheral veins in a clinical study unit. Meals were provided at 08.00, 12.30 and 17.30 h, and room lights were turned off between 22.00 and 24.00 h depending upon individual sleeping habits.

### 2.3. Collection and processing of blood samples

All surgical procedures were scheduled first of the day (beginning at 08.00 h). Surgical patients had blood sampled for 12 h via their *in situ* vascular catheters from first cannulation using a needle-free, closed-loop sampling system (Edwards VAMP. Edwards Life Sciences Corp., Irvine, CA. USA). Healthy controls were sampled from peripheral veins by either hand sampling or an automated blood sampling system [13]. Total serum cortisol, ACTH and seven inflammatory mediators (IL1*α*, IL2, IL4, IL6, IL8, IL10 and TNF*α*) were sampled at 10-minute intervals. Cortisol samples were collected in BD vacutainer SST Advance tubes (Becton, Dickinson and Company, Oxford, UK) and were processed immediately after centrifugation. Samples for ACTH were collected in chilled 2 ml EDTA tubes and stored on ice for a maximum of 60 min before centrifugation at 4°C and then stored at −80°C until assay. Total cortisol and ACTH were measured by solid phase, chemo-luminescent enzyme linked immunoassay (ECLIA) using the Cobas e602 modular analyser (Roche Diagnostics Ltd, West Sussex, UK). Measuring limits for the cortisol assay were 0.5–1750 nmol l^−1^ (intra- and inter-assay coefficients of variation (COV): 1.5–1.7% and 1.8–2.8%, respectively) and for the ACTH assay were 1.0–2000 pg ml^−1^ (intra- and inter-assay COV: 0.6–2.7% and 3.5–5.4%, respectively). Inflammatory mediators were collected in the same BD vacutainer SST advance tubes as cortisol. After centrifugation, aliquots were stored at −80°C until assay. Inflammatory mediators were assayed using the Luminex Multiplex system (ThermoFisher Scientific, Waltham, MA).

### 2.4. Statistical analysis

To characterize the dynamic responses elicited by surgery, we quantified the synchrony between ACTH and cortisol time series by means of non-stationary statistics such as time-dependent hormone ratios and their dynamic range, time-dependent cross-correlations, angle and instantaneous phase synchrony (IPS) [14,15]. We also calculated the time-lagged cross-correlation (TLCC) to quantify the strength of peak association (Spearman's *r*) and to estimate the time lag between ACTH and cortisol dynamics. A rolling window time-lagged cross-correlation (RWTLCC) was used to quantify the time lag when the peak association occurred for a range of elapsed time values from the beginning of the time series. The RWTLCC was digitally represented through a heat map, where vertical stripes emerge when the association between variables becomes stable over time and therefore their association is stationary. To estimate the potential effects of inflammatory mediators not accounted for in the model, we first performed a principal component analysis (PCA) on the trajectories of inflammatory mediators following CABG. Then the time-varying residual error between the model predictions and the cortisol trajectories was calculated. Following Z-score normalization, we extracted correlations between the model error and the inflammatory mediator dynamics. This allowed us to compare each cytokine time-varying trajectory with the error of predicted cortisol levels for each patient, and visually inspect where the model discrepancies with data could possibly be explained by cytokine dynamics. Since inflammatory mediators are likely to act together in regulating the HPA axis, we calculated correlations not only for cytokines IL6 and TNF*α* identified by the PCA, but also for IL8, IL10 and additive combinations of them.

### 2.5. Mathematical model

The model assumes a delayed (10 min) ACTH input to a hypothetical adrenal gland underpinning cortisol production (implicitly including peripheral conversion from cortisone), cortisol turnover, and the adrenal sensitivity to ACTH stimulation. It also assumes a two-compartment, open-loop architecture where each compartment accounts for fast and slow processes contributing to cortisol dynamics, respectively [16]. While ultradian pulsatility of ACTH and cortisol arises from a delayed negative feedback loop between the pituitary–adrenal system [17], using an open-loop architecture allows us to use one of the hormone data signals as a model input (ACTH), while studying the effects of that signal on the other hormone (cortisol). This has the advantage of reducing the number of parameters that would have been required to fit in a closed-loop model (and associated estimation error), while instead allowing for any additional parameters to distinguish between processes occurring at different timescales. The model was developed following an approach similar to existing models [10,18], and is represented by the equation:

$$\frac{dC(t)}{dt}=\frac{dC_{f}}{dt}+\frac{dC_{s}}{dt}=\;-\left(\frac{C_{f}}{\lambda_{f}}+\frac{C_{s}}{\lambda_{s}}\right)+(p_{f}+p_{s})\frac{A{\left(t-10\right)}^{m}}{K_{A}^{m}+A{\left(t-10\right)}^{m}}.$$

Cortisol dynamics *C*(*t*) are split in the model as the contribution of two compartments, *C*_*f*_ and *C*_*s*_, respectively accounting for fast and slow timescales for cortisol dynamics. In the model (figure 1*d*), parameters *p*_*f*_ and *p*_*s*_ account for fast and slow cortisol production respectively (including adrenal maximum secretory capacity and peripheral conversion from cortisone); *1*/*λ*_*f*_ and *1*/*λ*_*s*_ are the fast and slow cortisol turnover rates, respectively; *K_A_* is the adrenal sensitivity to ACTH stimulation; and *m* is the Hill coefficient denoting the steepness of the sigmoidal function used to represent the adrenal response to ACTH. *A* is the ACTH data. Overall, the model has six parameters, some of which were fixed at different stages in the study. The initial condition for the model is chosen by setting the fast compartment *C_f_* to quasi-equilibrium, then $C_{s}(0)=\;\max(C_{\mathrm{data}}(0)-C_{f}(0),0)$.

We performed computer simulations of the model and used an optimization algorithm to calibrate it to physiological data. ACTH data were an input and the ability of the model to accurately capture the cortisol output trajectories over 1 million sets of parameter values was assessed. Further details about the model optimizations can be found in the electronic supplementary material.

## 3. Results

### 3.1. CABG disrupts normal HPA rhythmicity and elicits a dynamic inflammatory response

Ten patients underwent isolated CABG surgery via median sternotomy (table 1). CABG can take place with or without cardiopulmonary bypass (CPB). Five cases had their surgery performed with the use of CPB and five did not. When CPB was used, the duration was 67 ± 20.7 min. Patients and controls were not matched for age, height and weight, but were broadly similar in these parameters. No patient suffered major complications and this was indicated in the median critical care and hospital length of stay, which were shorter than the UK median (3.2 and 7.2 days, respectively) [19,20].

**Table 1.** Demographic and operative data of study participants.

	controls (*n* = 3)	patients (*n* = 10)
age	54 ± 13.8 years	65 ± 6.2 years
height	180 ± 6.7 cm	173 ± 6.4 cm
weight	87.5 ± 13.7 kg	83 ± 10.7 kg
BMI	26.9 ± 2.1 kg m^−2^	27.8 ± 4.1 kg m^−2^
sampling duration	24 h	12 h
anaesthesia start time	—	8.26 h (± 22 min)
surgery start time	—	9.19 h (± 22 min)
surgery end time	—	12.15 h (± 60 min)
surgery duration	—	176 ± 58.2 min
critical care stay	—	3 (range 2–7) days
hospital stay	—	5 (range 4–8) days

All patients exhibited disrupted ACTH and cortisol rhythms following surgery, with hormone concentrations consistently increasing at a time of the day when the control patients' trend was to decrease (electronic supplementary material, figure S1). At its maximum (2 h post-surgery), mean ACTH levels increased approximately 10-fold compared to physiological levels before decreasing again during the last 6 h of sampling. By contrast, mean cortisol increased steadily from the end of surgery, approximately doubling mean physiological levels by the end of sampling (8 h post-surgery). The mean levels of inflammatory mediators IL6, TNF*α*, IL10 and IL8 exhibited dynamic responses as well, but these typically started during the second half of the surgery (figure 1*c*). IL2, IL1*α* and IL4 were not or inconsistently detected across patients (electronic supplementary material, figure S2).

In the healthy controls, ACTH and cortisol exhibited regular ultradian pulsatility (*T*_u_ = 2.5–3 h) with a circadian modulated amplitude (electronic supplementary material, figure S1). The area under the curve (AUC) for ACTH and cortisol between 08.00 h and 20.00 h (equivalent to the 12 h sampling time interval for patients) was consistent across controls, with AUC_ACTH_ = 196.65 ± 42.81 ng h l^−1^ and AUC_CORT_ = 2896.62 ± 221.04 nM h. During and after CABG, the dynamic responses of these hormones and inflammatory mediators varied across patients (electronic supplementary material, figure S2), with an almost sixfold increase in total ACTH secretion with respect to controls (AUC_ACTH_ = 1228.82 ± 831.59 ng h l^−1^) but only about a twofold increase in total cortisol secretion compared to controls (AUC_CORT_ = 6473.7 ± 1181.67 nM h). Time-series analysis of hormone profiles showed that CABG induces different ACTH/cortisol ratios compared to controls (electronic supplementary material, figures S1 and S3). The time-dependent Pearson correlation coefficient showed longer-lasting correlated and anti-correlated events between ACTH and cortisol compared to controls, while the angle and IPS showed a loss of phase coherence between ACTH and cortisol rhythms (electronic supplementary material, figures S1 and S3).

### 3.2. Identifying three distinct phenotypes of HPA axis activity following CABG

**Figure 2.**
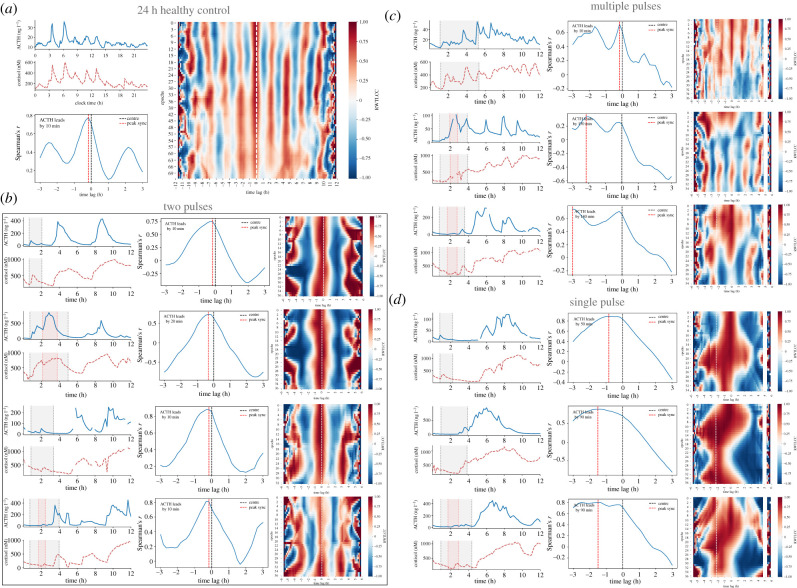
Dynamic responses of ACTH and cortisol during and after CABG (*n* = 10). (*a*) Ultradian rhythms with circadian modulated amplitude in one healthy control. While the time-lagged cross-correlation (TLCC) quantifies peak association between ACTH and cortisol, the rolling window TLCC heat map (RWTLCC) characterizes the phase synchrony across all epochs (elapsed time increases downwards). We use these techniques to identify three types of CABG-induced cortisol profile phenotype: (*b*) two pulses: characterized by two ACTH and cortisol pulses of supra-physiological amplitude and period, but without loss of phase synchrony; (*c*) multiple pulses: characterized by multiple ACTH and cortisol pulses of increased amplitude but near normal periodicity, with loss of peak association but no apparent loss of phase synchrony, and (*d*) single pulse: characterized by a single ACTH and cortisol pulse of supra-physiological amplitude, with loss of both peak association and phase synchrony. Grey (red) shaded areas in the hormone time-series indicate duration of CABG surgery (CPB).

The TLCC and RWTLCC showed that the healthy control group had a high peak association between ACTH and cortisol, with ACTH leading cortisol by a mean lag of *μ*_lag_ = 10 min which remained stable across all epochs (figure 2*a* and electronic supplementary material, figure S1). By contrast, the patients having cardiac surgery experienced different lags and degrees of phase synchrony loss between ACTH and cortisol. Combining the period, TLCC and RWTLCC allowed us to group the dynamic responses of the HPA axis into three categories (figure 2*b–d*) according to the type of dynamic dissociation between ACTH and cortisol:

— ***Two-pulse group.*** These patients showed two pulses of ACTH and cortisol with longer than physiological periodicity (*T*_u_ = 5–6 h) but preserving a near-physiological peak association (*μ*_lag_ = 13 min) and a stable phase synchrony across the entire 12 h long sampling (figure 2*b*, see patients 1, 5, 6 and 7 in electronic supplementary material, figure S2).
— ***Multiple-pulse group.*** These patients showed multiple pulses of ACTH and cortisol (*T*_u_ = 2 hrs) with strong peak dissociation (*μ*_lag_ = 106 min) and partial phase synchrony during a portion of the 12 h long sampling (figure 2*c*, see patients 4, 9 and 10 in electronic supplementary material, figure S2).
— ***Single-pulse group.*** These patients showed a single large excursion of ACTH and cortisol with strong peak dissociation (*μ*_lag_ = 77 min) and unstable phase synchrony across the entire 12 h long sampling (figure 2*d*, see patients 2, 3 and 8 in electronic supplementary material, figure S2).

### 3.3. A model of cortisol concentration accounts for the dynamic changes of the HPA axis following CABG

To investigate the origin of the dynamic dissociation between ACTH and cortisol observed across the three groups of patients (two pulses, multiple pulses and single pulse), we fitted the model to identify optimal parameter values for each group (electronic supplementary material, table S1). We characterized the differences between controls and patients, and between patient groups with distinctive cortisol phenotypes, by means of violin plots representing the distributions of optimal parameter values that fit the model to data. The fits to each patient group were marginally improved compared to the fits to all CABG patients pooled together, with an error as low as $\tilde{\varepsilon }=0.1$ for the two-pulses group, $\tilde{\varepsilon }=0.08$ for the multiple-pulses group, and $\tilde{\varepsilon }=0.1$ for the single-pulse group (electronic supplementary material, table S2). The shapes of distributions of parameter values associated with fast processes (*p*_*f*_, *λ*_*f*_) were also very similar across all CABG groups and between these and the control group (electronic supplementary material, figure S4). For parameters associated with slow processes, the cortisol production rate *p*_*s*_ and half-life *λ*_*s*_ were predicted to remain within the same dynamic range (although slightly higher) as controls for the two-pulses and multiple-pulses CABG groups (electronic supplementary material, table S1). The shape of the *λ*_*s*_ distribution tended towards higher values in these patient groups. By contrast, the single-pulse CABG group was predicted to have a higher cortisol production rate *p*_*s*_, but a lower half-life *λ*_*s*_. The adrenal sensitivity *K*_*A*_ was similar between the control and the two-pulses CABG group but was predicted to have lower values (implying higher adrenal sensitivity) for the single-pulse and multiple-pulses groups (electronic supplementary material, figure S4). The estimated distributions of parameter values for the two-pulse group maintained similar statistical moments to the healthy controls, only changing the shape of the *λ_s_* distribution. The distributions for the multiple-pulses group were similar to the two-pulse group, except that the model also predicts an increased adrenal sensitivity (lower *K_A_*). Finally, the distributions for the single-pulse group showed that not only is the adrenal sensitivity increased, but also cortisol secretion *p_s_* and its turnover rate 1/*λ_s_* are as well. The dynamic range and shape of the distribution of *K_A_* predicted for the two-pulses CABG group was very close to controls, suggesting that the adrenal sensitivity to ACTH is the same for these patients.

**Figure 3.**
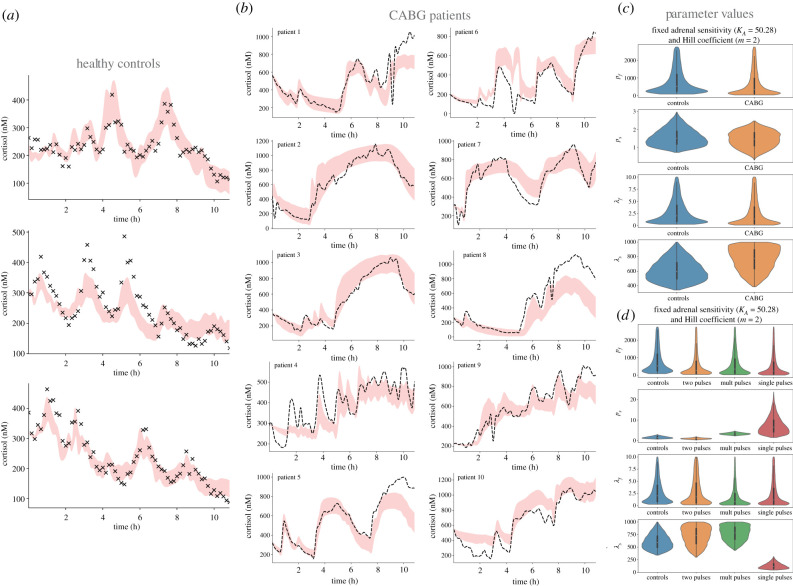
Mathematical model predictions versus data. The model was calibrated using (*a*) healthy control data (black dots), and validated against (*b*) CABG patient data (broken lines). In both cases, the shaded red curves indicate model predictions. (*c,d*) Distributions of best parameter fits with fixed adrenal sensitivity (*K*_*A*_ = 50.28) and fixed Hill coefficient (*m* = 2) for controls, all patients grouped together, and patients grouped by their type of chronodisruption.

To narrow the origin of the dynamic dissociation between ACTH and cortisol, we assumed that CABG surgery does not markedly change the adrenal sensitivity to ACTH stimuli. This stems from the knowledge that such changes are unlikely to take place at the timescale of hours after surgery, but may result from long-term critical illness [21,22]. To do this, we fixed the adrenal sensitivity parameter to the median value estimated for controls (*K*_*A*_ = 50.28) and identified the distributions of parameter values as before (electronic supplementary material, table S3). The model predicted low error trajectories for both the controls ($\tilde{\varepsilon }=0.07$, figure 3*a*) and the CABG group ($\tilde{\varepsilon }=0.14$, figure 3*b*). The optimal parameter sets, when comparing the control and CABG group, showed these to be similar. The exception was the shape of the distribution for the half-life parameter *λ*_*s*_, which tended toward higher values (figure 3*c*).

Next, we investigated the model predictions for each CABG group under the assumption of fixed adrenal sensitivity. The shapes of distributions for optimal parameter values associated with fast processes (*p*_*f*_, *λ*_*f*_) were again similar across all CABG groups and the control group (figure 3*d*). For parameters associated with slow processes, the cortisol production rate *p_s_* and half-life *λ_s_* were predicted to remain within the same dynamic range (albeit slightly higher) as controls for the two-pulses and multiple-pulses CABG groups (electronic supplementary material, table S4), with the shape of the *λ*_*s*_ distribution leaning towards higher values in these patient groups. Consistent with the variable *K*_*A*_ scenario, the single-pulse CABG group was again predicted to have a higher cortisol production rate *p*_*s*_, but a shorter half-life *λ*_*s*_.

### 3.4. Inflammatory mediators may underpin the dynamic changes in the interaction of ACTH and cortisol following CABG

**Figure 4.**
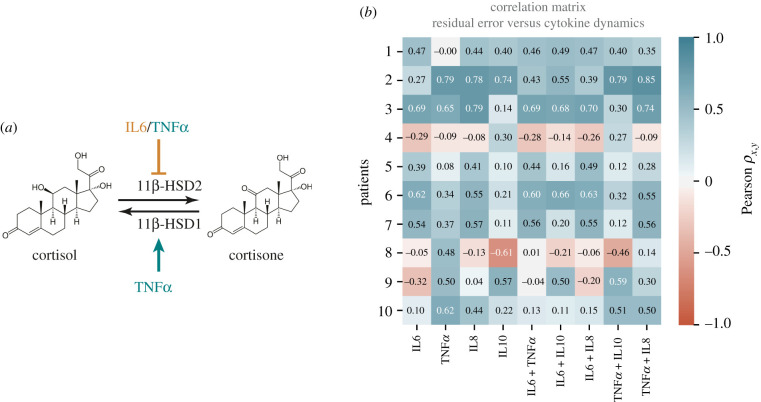
Correlation between model predictions error and cytokine dynamics. (*a*) Tissue-level regulation of cortisol availability by inflammatory mediators targeting 11β-HSD1/2. (*b*) Correlation matrix between the model residual error and the dynamics of inflammatory mediators for each patient. Patients in the two-pulses group: 1, 5, 6 and 7. Patients in the multiple-pulses group: 4, 9 and 10. Patients in the single-pulse group: 2, 3 and 8.

Consistent with known effects of cytokines on the HPA axis at the cellular level [6,7], we hypothesized that IL6 and TNF*α* might be the key cytokines mediating slow regulation of cortisol metabolism (figure 4*a*). We systematically explored the potential regulatory effects of IL6, TNF*α*, IL8, IL10 and combinations of them on explaining discrepancies between our model predictions and observed cortisol trajectories in CABG patients (electronic supplementary material, figure S5). This is summarized in figure 4*b*, where each patient's correlation between their predicted cortisol trajectory error and inflammatory mediators are represented as a row in a correlation matrix heat map. Patients 2, 3 and 8 (belonging to the single-pulse group) showed some of the highest positive (patients 2 and 3) and negative (patient 8) correlation coefficients. In contrast, patients 1, 5, 6 and 7 belonging to the two-pulse group showed positive but weak correlations with inflammatory mediators, while the remaining patients belonging to the multiple-pulse group showed a mix of low positive, low negative and near null correlations.

## 4. Discussion

A maladaptive response to acute systemic inflammation underlies most of the morbidity and mortality from major surgery [23] and critical illness (most notably sepsis [24] and more recently COVID-19 [25]). Therefore, understanding the mechanisms that modulate this is key to designing clinical diagnostics and interventions that reduce the risk of patient death and morbidity. The HPA axis is one such system that is thought to overall counterbalance inflammation—although this has been difficult to elucidate due to the multiple levels of interaction and sites of feedback between the two systems. High-frequency blood sampling techniques and mathematical models have advanced our understanding of the interaction between the two systems, but have not previously been combined in humans during clinical care [10,26]. We have previously shown that patients both after straightforward CABG [4] and who are critically ill [10] show continued interaction between their pituitary and adrenal glands. This study classifies and quantifies the differences and postulates the physiological mechanisms underlying them. On the other hand, previous mathematical models have been used to investigate the mechanisms of acute stress responses typically associated inflammation, albeit in a purely theoretical way or in combination with experiments in rodents [18,26,27]. Using a combination of high-frequency blood sampling and statistical analysis, this study has shown that at least three distinct phenotypes of cortisol dynamic profiles emerge after cardiac surgery: single pulse, two pulses and multiple pulses. The mechanisms underpinning these three different phenotypes were inferred using mathematical modelling, with parameter estimations used to predict the contribution of distinct cortisol control mechanisms in patients. Lastly, we identified that inflammatory mediators may only be important in one pattern of HPA axis activity, paving the way to identify those most at risk of serious harm from inflammation.

To our knowledge, this is the first study combining high-frequency sampling of patients after cardiac surgery together with statistical analysis and mathematical modelling. We postulated a two-compartment mathematical model of cortisol activity that discriminates between fast and slow processes, with model parameters corresponding to the adrenal sensitivity to ACTH, cortisol production (a contribution of adrenal secretory capacity and peripheral conversion from cortisone), and cortisol turnover and metabolic rates (arising from degradation and inactivation into cortisone). We determined the models with the best fits by assuming a similar adrenal sensitivity between patients undergoing CABG and controls. During fitting, only the parameters associated with fast processes remained invariant between the control and patient groups. This suggests that CABG may not affect cortisol regulatory processes associated with short timescales (e.g. rapid cortisol synthesis and distribution out of the central (plasma) compartment), but only affects slower processes such as enzymatic degradation. When examining the slow parameters, the model predicted that patients in the single-pulse group had a greater slow cortisol production rate and higher slow turnover rate compared to controls and other patient groups. The single-pulse group also had the highest dynamic dissociation between ACTH and cortisol. The model predicted that patients in the two-pulses group had a similar cortisol production rate and an increased slow turnover rate compared to controls. This group also had the lowest dynamic dissociation between ACTH and cortisol following surgery. Lastly, the model predicted that patients in the multiple-pulses group had an increased slow cortisol production rate and an increased slow turnover rate when compared to controls. This group also had a dynamic dissociation between ACTH and cortisol falling in the middle of the other two phenotypes. Taken together, these results suggest which mechanisms may underpin the three different phenotypes of cortisol activity observed in patients undergoing CABG. Our model also suggests that inflammatory mediators do not play a significant role in regulating circulating levels of cortisol in *all* patients having CABG within the 12 h following surgery, a result that triangulates with findings using a meta-analysis of clinical studies [28]. However, in some cases, inflammatory mediators may cause significant disruption to HPA axis dynamics, leading to a large, sustained pulse of cortisol.

There are several limitations to consider when interpreting the results of the mathematical model. While the fast and slow timescale separation in the model has been described previously [10,16], the optimization is agnostic with respect to the physical mechanisms underlying this separation and only attempts to minimize the cost function. Therefore, some of the fast mechanisms of cortisol dynamics are captured by the slow variable of the model and vice versa, but this is likely to be minimal. On the other hand, we consider ranges of parameter sets that fit the data well, rather than only considering the single best fit parameter set. This is to minimize concerns of overfitting the data. This means that we can only assess the difference between groups, but not assume that a single group in isolation provides specific estimates of parameter values. Our sample size of patients and healthy participants was small, so we cannot be sure that we have captured the full range of possible HPA axis response patterns that occur after cardiac surgery. It also means we were unable to fully investigate the patient and operative factors that may cause or are associated with the different patterns of physiological processes. One of the differences we were unable to capture was the effect of female sex. Sex hormones are well known to affect ultradian patterns of hormone secretion [11,12] as well as adrenal sensitivity [27] and therefore we did not include females in this study. This was to trade off the issue of heterogeneity in the study population and ensure an achievable sample size in the context of a sampling approach that was labour intensive. However, this does mean that currently, the results are only applicable to men undergoing CABG. Women are at a higher risk of short-term mortality after cardiac surgery [29,30]. It is unclear how much of this difference is physiological and how much is organizational. Comparison of male and female differences in HPA-axis physiology may provide some insight to this. We envisage that novel blood-free biosampling technologies [31] will facilitate sampling a larger number of patients with more heterogenous characteristics. This will allow a better characterization of CABG phenotypes as well as investigating the effects of cytokines over a longer timeframe.

Our model does not consider inflammatory cytokines either as state variables or model inputs (as was the case with ACTH). Nor does it consider any explicit modulation by inflammatory mediators on ACTH—our open-loop model architecture assumes such modulation is already accounted for in the resulting ACTH dynamics. Instead, we try to capture potential time-varying modulations of cortisol using parameter changes that remain fixed over time. This could explain the increased error in the CABG patient fits relative to the control fits. This is to be expected given the inflammatory response is only present in CABG patients. However, including inflammatory cytokines as model inputs would not only require additional parameters and therefore increase the risk of overfitting, but would also require detailed knowledge of the interactions between the HPA axis and inflammatory pathways. Although some advances have been made in modelling these interactions [18,26], uncovering the network architecture between these pathways after major surgery and in critical illness has so far not been achieved. To do so will require a larger cohort of patients and a combination of mathematical modelling and machine learning techniques [32].

## 5. Conclusion

We have been able to show that there is not a simple graded HPA response to cardiac surgery. There are major dynamic changes involving not only the concentration of ACTH and cortisol but also their pattern of secretion. Using novel mathematical techniques, we show that CABG patients exhibit three different patterns of HPA axis response, which reflect different underlying physiological changes in adrenal sensitivity, cortisol production and turnover. Inflammatory mediators appear to be driving changes in only one of these patterns (a single pulse) despite being postulated as the likely cause for the elevated cortisol seen in *all* types of systemic inflammation [18,33]. We suggest that the different patterns we have observed may represent different ‘severities’ of response to the surgery. An adequately powered study is now required to investigate whether and how these patterns are correlated with clinical outcomes. This will be critical to establish whether the patterns could be used for risk stratification after surgery. This study also shows that the existing model of corticosteroid physiology used for diagnosis and prognosis after major surgery and in critical illness [34] may only represent mean values within a population rather than the responses of individuals or groups of individuals. Improved diagnostics based on individual or subgroup responses is likely to lead to greater precision in diagnosis and more targeted interventions.

## Data Availability

The datasets and modelling code (implemented in Python) supporting the conclusions of this article are available in Github: https://gitlab.com/ezavala1/CABG_phenotypes. The full model and optimization pipeline was implemented in MATLAB and is available at https://github.com/dgalvis/heart_surgery_model. The data are provided in electronic supplementary material [35].
